# Simulating an intravenous-to-oral cefotaxime-to-cefditoren/cefuroxime switch: an *in vitro* pharmacodynamic approach against selected *Streptococcus pneumoniae* strains with defined susceptibility profiles

**DOI:** 10.3389/fphar.2026.1770715

**Published:** 2026-08-24

**Authors:** Natalia González, Eva Manceras Jiménez, María José Giménez, Luis Alou, Pilar Coronel, Elena Gómez-Rubio, María Luisa Gómez-Lus, David Sevillano

**Affiliations:** 1 Microbiology Area, Medicine Department, School of Medicine, Universidad Complutense de Madrid, Madrid, Spain; 2 Department of Physiotherapy, Faculty of Medicine, Health and Sport, Universidad Europea de Madrid, Madrid, Spain; 3 Scientific Department, Meiji Pharma Spain, Madrid, Spain

**Keywords:** cefditoren, cephalosporin, IV-to-Oral switch therapy, PK/PD system, *Streptococcus* pneumoniae

## Abstract

**Introduction:**

The lack of oral equivalent for third-generation IV cephalosporins challenges sequential cephalosporin therapy for pneumococcal infections. This *in vitro* study combined the previously reported pharmacokinetics of IV cefotaxime (CTX) and oral cefditoren (CDN) or cefuroxime (CXM) to simulate an intravenous-to-oral switch and explore the pharmacodynamic activity against *S. pneumoniae*.

**Methods:**

We used an *in vitro* PK/PD system to expose four *Streptococcus pneumoniae* strains (serotype/MIC_CTX_ mg/L): 1S (23B/0.25), 2S (6A/0.5), 3I (35B/1), and 4I (11A/1), to free serum concentrations simulating an initial IV dose of 1,000 mg CTX, followed by two oral doses of 400 mg CDN bid (CTX-to-CDN) or 500 mg CXM bid (CTX-to-CXM) over 36 h. Reference regimens included a single-dose CTX regimen and a full 1,000 mg bid CTX regimen (CTX-IV). Bacterial growth (K) was used as a control.

**Results:**

Against strain 1S, both oral cephalosporins sustained bactericidal activity in the model, similar to the complete CTX-IV regimen. For strain 2S, CTX-to-CDN maintained bactericidal efficacy comparable to CTX-IV, while CTX-to-CXM showed with counts at 36 h similar to initial inoculum. Against strain 3I, CTX-to-CDN exhibited a bacteriostatic effect, whereas CTX-to-CXM was equivalent to the growth control. Strain 4I was associated with regrowth in both sequential regimens. The CTX-IV regimen was bacteriostatic against strains 3I and 4I.

**Discussion:**

Overall, despite the limitations of the proposed model in reproducing the antimicrobial activity of agents with high plasma protein binding, such as CDN, greater *in vitro* pharmacodynamic activity was observed with CDN than with CXM in the simulated sequential regimens against the four strains tested, particularly against CTX-susceptible strains. These findings warrant confirmation in clinical studies before any therapeutic recommendation could be made.

## 1 Introduction

Bacterial respiratory infections remain a leading cause of morbidity and mortality worldwide, with *S. pneumoniae* continuing to be the main target of empirical antibiotic treatment (Cantón et al., 2022). Although published reports highlight the declining incidence of *Streptococcus pneumoniae* as etiological agent of community-acquired pneumonia (CAP), particularly after introduction of conjugate vaccines, pneumococci continue to lead among the isolated bacterial species (Shoar and Musher, 2020). In exacerbations of chronic obstructive pulmonary disease (COPD), *Haemophilus* influenzae may be more prominent, but antibiotic therapy must also cover the other two most common bacterial pathogens, *S. pneumoniae* and *Moraxella catarrhalis* (Labaki and Rosenberg, 2020). For patients with CAP, approximately 40%–50% required hospitalization (Almirall et al., 2017; Martin-Loeches et al., 2023; Tsoumani et al., 2023). In these cases, rapid initiation of empirical intravenous (IV) therapy is crucial to reduce mortality. The American Thoracic Society/Infectious Diseases Society of America (ATS/IDSA) recommends a β-Lactam/macrolide combination as the standard empirical therapy for severe CAP (Metlay et al., 2019).

Sequential antibiotic therapy—defined as the planned transition from IV to oral treatment—offers several advantages in managing CAP, among others, reduced hospital stays, lower healthcare costs, and improved patient comfort and adherence (Martin-Loeches et al., 2023; McCarthy and Avent, 2020; Metlay et al., 2019). Moreover, a timely switch from IV to oral antimicrobial therapy is widely recognized as an effective antimicrobial stewardship strategy (Harvey et al., 2023). A short course of IV therapy for 48–72 h can be converted to appropriate oral formulations once patients are clinically stable (Cantón et al., 2022; Harvey et al., 2023; McCarthy and Avent, 2020; Metlay et al., 2019; Sánchez Fabra et al., 2021). In switching from parenteral to oral antibiotics, either the same agent or the same drug class should be used (Metlay et al., 2019). However, conversion from IV to oral cephalosporin therapy is not as straightforward as with other antibiotics as fluoroquinolones, since the third-generation IV cephalosporins used for empirical treatment of CAP lack equivalent oral formulations. Clinicians must carefully consider drug pharmacokinetics, bioavailability, and patient-specific factors to ensure therapeutic continuity and efficacy. Lack of an oral equivalent for the IV antibiotic administered to the patient is a recognized barrier to switching from IV to oral therapy (Deshpande et al., 2023). The challenge is particularly significant when the infectious microorganism cannot be isolated, a scenario observed in more than 50% of cases (Jain et al., 2015). As a result, sequential therapy is often initiated too late, and only in a small proportion of patients for whom it would be advisable (Arteche-Eguizabal et al., 2022; Deshpande et al., 2023; Kimura et al., 2020; Sánchez Fabra et al., 2021).

In this context, cefditoren (CDN), a third-generation oral cephalosporin, characterized by an *in vitro* antimicrobial potency equal to or greater than cefotaxime (CTX) against *S. pneumoniae* (Sempere et al., 2022a) could represent a strong candidate for therapeutic transition after initial IV cephalosporin treatment (Cantón et al., 2022; Rodríguez-Leal et al., 2024). Extensive research on the pharmacokinetics/pharmacodynamics (PK/PD) properties of CDN against penicillin-resistant pneumococcus has been conducted using pharmacodynamic simulations (Cafini et al., 2008a; Cafini et al., 2008b; Giménez et al., 2018). Pharmacodynamic simulations allow for the integration of drug concentration-time profiles with bacterial kill kinetics, offering a mechanistic understanding of therapeutic efficacy. The results obtained from PK/PD models provide robust support for clinical decision-making, particularly when treating infections caused by resistant microorganisms (Pereira et al., 2022). This is especially relevant for *S. pneumoniae*, where drug-resistant isolates account for more than two out of five infections in the USA (Centers for Disease Control and Prevention, 2024).

This pharmacodynamic simulation aimed to explore class-concordant sequential antibiotic regimens against *S. pneumoniae* when initial therapy involves CTX, an IV third-generation cephalosporin. Accordingly, the PK/PD activities of CDN and cefuroxime (CXM), two widely used oral cephalosporins and clinically relevant step-down options, were compared with the continued intravenous therapy (CTX-IV). To bridge the gap between *in vitro* assays and real-world clinical application, all experiments were conducted considering only pharmacokinetic data from regimens already approved for human treatment, to address the challenge encountered by clinicians when choosing and oral treatment for sequential therapy. However, it should be noted that, for the model to be feasible, treatment durations had to be adapted to the *in vitro* system and thus do not equal those used in clinical practice.

## 2 Materials and methods

### 2.1 Antimicrobial agents

CTX and CXM powders (sodium salt) were purchased from Sigma-Aldrich (Merck KGaA, Darmstadt, Germany). CDN powder (sodium salt) was kindly provided by Meiji Pharma Spain (Madrid, Spain). For the assays, antibiotic stock solutions were freshly prepared at a concentration of 1 mg/ml in sterile water.

### 2.2 Bacterial strains

Four clinical isolates of *S*. *pneumoniae*, strain 1S, 2S, 3I and 4I, belonging to common circulating serotypes with a MIC_CTX_ ranging from 0.25 to 1 mg/L, were selected from the Spanish Pneumococcal Reference Laboratory collection at Instituto de Salud Carlos III (Madrid, Spain). Penicillin and amoxicillin MICs were 0.12 and 0.25 mg/L for strain 1S, 2 and 4 mg/L for strains 2S and 3I, and 2 and >8 mg/L for strain 4I, respectively. The serotypes and susceptibilities to CTX are summarized in Table 1. According to EUCAST breakpoints for CTX (S (susceptible): ≤0.5; R (resistant): >2) (The European Committee on Antimicrobial Susceptibility Testing (EUCAST), 2025), strains 1S and 2S were susceptible while strains 3I and 4I were intermediate. Based on CLSI breakpoints (S: ≤1; R:≥4) (Clinical and Laboratory Standards Institute, 2025), all four strains were susceptible to CTX.

**TABLE 1:** Susceptibility of the *Streptococcus pneumoniae* strains used in this study.

Antibacterial agent	MIC/MBC (mg/L) for strain (serotype)			
	1S (23B)	2S (6A)	3I (35B)	4I (11A)
Cefotaxime	0.25/0.25	0.5/0.5	1/1	1/2
Cefditoren	0.06/0.06	0.12/0.12	0.25/0.25	0.5/0.5
Cefuroxime	0.12/0.12	1/1	2/4	4/4

### 2.3 Antibiotic susceptibility testing

MICs and MBCs were determined by broth microdilution following CLSI recommendations (Clinical and Laboratory Standards Institute, 2024), using Todd-Hewitt broth (BD, Becton, Dickinson and Company, Franklin Lakes, NJ) supplemented with 0.5% yeast extract (BD) (THB-YE). Antibiotics were tested at final concentrations ranging from 0.0035 to 8 mg/L. MICs were determined by visual reading after 18–20 h of incubation at 37 °C in a 5% CO_2_. *Streptococcus pneumoniae* ATCC 49619 was included in each run as the reference quality-control strain and obtained MIC values fell within acceptable ranges across all experimental batches. Subsequently, 10 μL from each well was plated on Columbia agar (BD) to determine the MBC. All determinations were performed in triplicate on separate days. Modal values were considered.

Additionally, MIC and MBC determinations were performed using a high-density inoculum of approximately 10^7^ CFU/mL following the same broth microdilution procedures.

### 2.4 *In vitro* PK/PD system

A previously described two-compartment *in vitro* PK/PD system (González et al., 2011) was used to assess the antibacterial effect of the free serum concentration dosing profiles of two cephalosporin IV-to-oral switch regimens against the selected *S*. *pneumoniae* strains.

Briefly, the system (Supplementary Figure S1) consists of a dilution reservoir, a central flask, and a hollow-fiber bioreactor (FX 50 dialyzer, Fresenius Medical Care, Bad Homburg v. d. Höhe, Germany), all interconnected by a continuous loop of silicone tubing. The central flask, the hollow fiber, and the interconnection tubing with the bioreactor together compose the central compartment (PK compartment). The extra-capillary space of the bioreactor constitutes the peripheral compartment (infection site), where the confined bacteria are exposed to the changing antibiotic concentrations from the central compartment. The entire system is maintained at a constant temperature of 37 °C.

The operational procedure of the system has been described in detail elsewhere (González et al., 2011). Before each experiment, the system was filled and primed with THB-YE. Computerized peristaltic pumps (Masterflex L/S, Cole-Parmer, Chicago, IL) pumped nutrient broth from the dilution reservoir to the central flask and through the central compartment. The central flask is connected to a collecting vessel for overflow to maintain a constant volume of distribution in the system (350 ml). Antimicrobial agent was delivered directly into the central flask using a programmable dosing pump (Gilson Inc, Middleton, WI).

The rates of antibiotic infusion and nutrient broth dilution-elimination in the central compartment were set according to the absorption and elimination constants of the antimicrobial agent. All system flow rates were controlled using WinLIN software, version 3.2 (Cole-Parmer).

The high surface area-to-volume ratio of the bioreactor (>200 cm^2^/ml) ensures a quick and even distribution of nutrients and antimicrobials between both compartments.

### 2.5 Simulated dosing regimens

The free concentration/time profiles of cephalosporin IV-to-oral switch regimens, consisting of an initial IV dose of 1,000 mg of CTX followed by two oral doses of either 400 mg of CDN every 12 h (CTX-to-CDN) or 500 mg of CXM every 12 h (CTX-to-CXM), were simulated over a 36-h period.

The proposed sequential regimens were compared to two reference regimens: (i) the full CTX IV regimen (CTX-IV: free concentration/time profile of three consecutive IV doses of 1,000 mg of CTX every 12 h) as the clinical reference for efficacy and (ii) a single-dose of CTX (CTX-to-K: free concentration/time profile of an initial IV dose of 1,000 mg of CTX followed by two consecutive sterile water doses every 12 h) as a pharmacodynamic control of the residual effect of the initial IV administration. Additionally, bacterial growth control was included (K: three consecutive sterile water doses given every 12 h).

For CTX, the free-drug parameters derived from published data (Esmieu et al., 1980) and the prescribing information for a 1,000 mg single dose assuming a mean protein binding of 40% (CIMA- Centro de información online de medicamentos de la AEMPS, 2025b), were targeted using a series of monoexponential elimination rates- 12.14 mL/min for 1 h (alpha half-life) followed by 4.04 mL/min for 11 h (beta half-life)- that mimic the multiexponential decay of CTX concentrations occurring in humans following IV dosing. Free-drug parameters for single doses of 400 mg of CDN and 500 mg of CXM, assuming mean protein binding values of 88% (CIMA - Centro de información online de medicamentos de la AEMPS, 2025a) and 50% (CIMA- Centro de información online de medicamentos de la AEMPS, 2025c), were targeted using flow rates set to 2.70 mL/min and 3.24 mL/min, respectively, to simulate their terminal elimination phase. CTX and sterile water were administered as a 30 s bolus. Oral cephalosporins were infused at a constant rate of 0.03 mL/min from time 0 to target T_max_, following a zero-order absorption kinetic. Throughout this phase, the dilution-elimination pumps remained continuously active to simulate physiological clearance. To ensure the targeted *f*C_max_ was precisely reached at T_max_, the infusion rate was mathematically adjusted using a one-compartment model for zero-order drug input with simultaneous first-order elimination, accounting for the drug loss due to continuous clearance during the absorption phase. For bacterial growth controls, the elimination constant of the cephalosporin with the longest half-life was adapted.

The target pharmacokinetic parameters, including the free maximum drug concentration in serum (*f*C_0_ or *f*C_max_), time to *f*C_max_ (T_max_) and elimination half-life (T_1/2_) are presented in Table 2.

**TABLE 2:** Pharmacokinetic parameters obtained for single-dose administration in the *in vitro* PK/PD model.

Pharmacokinetic parameter	Cefotaxime		Cefditoren		Cefuroxime	
	IV 1000 mg q12 h		Oral 400 mg q12 h		Oral 500 mg q12 h	
	Target^a^	Experimental^d^	Target^b^	Experimental^d^	Target^c^	Experimental^d^
C_max_ (mg/L)	102	N/A	4.1	N/A	7.7	N/A
*f*C_max_ (mg/L)	61.2	59.3 ± 1.6	0.5	0.5 ± 0.0	3.8	3.9 ± 0.0
T_max_ (h)	N/A	N/A	2.5	2.5 ± 0.0	2.4	2.4 ± 0.0
T_1/2_ alfa (h)	0.33	0.33 ± 0.02	N/A	N/A	N/A	N/A
T_1/2_ beta (h)	1	1.03 ± 0.02	1.5	1.50 ± 0.09	1.25	1.24 ± 0.06
*f*AUC_0-12_	N/A	36.5 ± 0.8	N/A	1.7 ± 0.0	N/A	10.7 ± 0.3
Calculated *f*T_>MIC_ (%)						
Strain 1S	49.6		57.8		>60	
Strain 2S	42.4		40.6		35.5	
Strain 3I	32.6		23.1		20.3	
Strain 4I	32.6		∼0.00		∼0.00	

T_1/2_ alpha: post-infusion elimination half-life. T_1/2_ beta: terminal elimination half-life.

^a^ Taken from references 26,27.

^b^ Taken from reference 28.

^c^ Taken from reference 29.

^d^ Experimental parameters are expressed as mean ± standard deviation (SD) of two independent experiments in uninfected bioreactors.

Analytical precision (% CV) and percent error relative to target (% error) for experimental pharmacokinetic parameters were as follows: for cefotaxime, *f*C_max_ (2.70% and 3.10%), T_1/2_ alfa (6.06% and 0.00%) and T_1/2_ beta (1.94% and 3.00%); for cefditoren, *f*C_max_ (0.00% and 0.00%), T_max_ (0.00% and 0.00%) and T_1/2_ beta (6.00% and 0.00%). For cefuroxime, *f*C_max_ (0.00% and 2.63%), T_max_ (0.00% and 0.00%) and T_1/2_ beta (4.84% and 0.80%).

### 2.6 Drug concentration assay and observed pharmacokinetic

Targeted concentrations were confirmed in two independent experiments using uninfected bioreactors following single dose of CTX (1,000 mg IV), CDN (400 mg oral), and CXM (500 mg oral).

Concentrations were determined by bioassay as previously described (González et al., 2011). Briefly, aliquots of standard (prepared in THB-YE) and samples were dispensed into bored wells on antibiotic medium no. 1 agar (BD) plates containing the indicator organism. When necessary, test samples were diluted in THB-YE to ensure concentrations fell within the standard curve range.

Plates were incubated for 24 h at 37 °C, and inhibition zone diameters were measured. Concentrations were determined by interpolation from the linear regression of standard curves using GraphPad Prism software v.8 (GraphPad Software, San Diego, CA). All samples were assayed in triplicate to ensure analytical reproducibility.

The indicator organisms and assay parameters were as follows: *Morganella morganii* ATCC 8076H was used for CTX (range: 0.25–40 mg/L; limit: 0.06 mg/L) and CDN (range: 0.03–4 mg/L; limit: 0.015 mg/L). *Bacillus subtilis* spores were used for CXM (range: 0.4–8 mg/L; limit: 0.25 mg/L). The correlation coefficient (r) for the standard curve within the range was >0.99.

Inter-day and intra-day coefficients of variations (%CV) were <3% (internal controls; 3 and 30 mg/L) for CTX, <2.78% (internal control; 0.75 mg/L) for CDN, and <5% (internal control; 1 mg/L) for CXM.

Pharmacokinetic parameters were calculated from the concentration-time curves using a noncompartmental approach. C_0_ for IV administration was estimated by log-linear regression from the first three observed concentration/time points. *f*C_max_ and T_max_ for oral doses were derived directly from observed data. The 12-h free area under the curve (*f*AUC_0-12_) was calculated using the log-trapezoidal rule. All analyses were conducted with Certara Phoenix 8.0 series software (Certara, Radnor, PA).

The time that free antibiotic concentrations exceeded the MIC (*f*T_>MIC_), expressed as a percentage of the dosing interval (0–12 h), was calculated from observed data using the pharmacodynamic data analysis module of Certara Phoenix software.

During the 36-h infected dynamic simulations, drug exposure to the bacterial inoculum was confirmed by collecting targeted spot samples at critical time points across all regimens. For CTX kinetic confirmation, samples were drawn at 0.25 h post-dose across all CTX-containing regimens, as well as at 6 h post-second dose in the reference CTX-IV regimen. For CDN and CXM kinetic confirmation in oral switch regimens (CTX-to-CDN and CTX-to-CXM), samples were collected at the target T_max_ of both the second and third oral doses, and at 6 h post-second dose. Random samples from at least two independent kinetic simulations per regimen and strain (104 samples tested in total) were quantified in triplicate by bioassay, as described above. Given the rapid clearance of CTX (T_1/2_∼1 h), residual concentrations 12 h post-dose were negligible, making assay interference during the oral phase unexpected.

### 2.7 Measurement of antibacterial effect

Test strain grown overnight on Columbia agar, were suspended in THB-YE and incubated in a static water bath at 37 °C for 5 h to a final density of 5–7x10^8^ CFU/mL (OD_490_ = 0.95–1.20). A 30 ml of this standardized suspension was inoculated into the peripheral compartment 1 h before starting the antimicrobial administration. For quantification of viable microorganisms, samples were obtained from the system at 0, 2, 4, 8, 10, 12, 14, 16, 18, 22, 24 and 36 h.

Undiluted and diluted (1:10–1:100,000) aliquots were plated on Columbia agar and incubated for 24 h at 37 °C with 5% CO_2_ before enumeration of viable microorganisms using an automated colony counter (BioMerieux, Marcy-l'Étoile, France). Results were expressed in log CFU/mL, with a detection limit of 1.7 log (50 CFU). Experiments were performed at least three times on different days.

The primary endpoint of the antimicrobial effect was the mean change in bacterial counts (in log CFU/mL) relative to initial inoculum, at 12, 24, or 36 h. Positive values denoted an increase in viable counts, while negative values indicated a reduction. Bactericidal activity was defined as 3 log killing of organisms compared to the inoculum.

### 2.8 Statistical analysis

Mean bacterial count changes (in log CFU/mL) relative to initial inoculum at 12, 24, and 36 h were evaluated separately for each strain and time point using one-way analysis of variance (ANOVA) followed by Tukey’s post-hoc test for multiple comparisons across treatment regimens. Target and observed pharmacokinetic parameters and, observed concentrations across the simulated regimen versus single-dose experiments were compared using the one-sample t-test Prediction accuracy was additionally quantified by calculating the percentage error. A P value of 0.05 was used for statistical significance. All statistical analyses were performed using GraphPad Prism software.

## 3 Results

MIC/MBC values (mg/L) for CDN and CXM are shown in Table 1. According to CLSI breakpoints for CXM (S: ≤1; R: ≥4), strains 1S and 2S were susceptible, strain 3I intermediate and strain 4I resistant to CXM. By applying EUCAST oral breakpoints (S: ≤0.25; R: >0.25), strains 2S, 3I and 4I were resistant to CXM. Currently, no breakpoints have been established for cefditoren to interpret susceptibility results. MIC and MBC values remained unchanged when using the high-density inoculum equivalent to that employed at the onset of the pharmacodynamic simulation.

**FIGURE 1:**
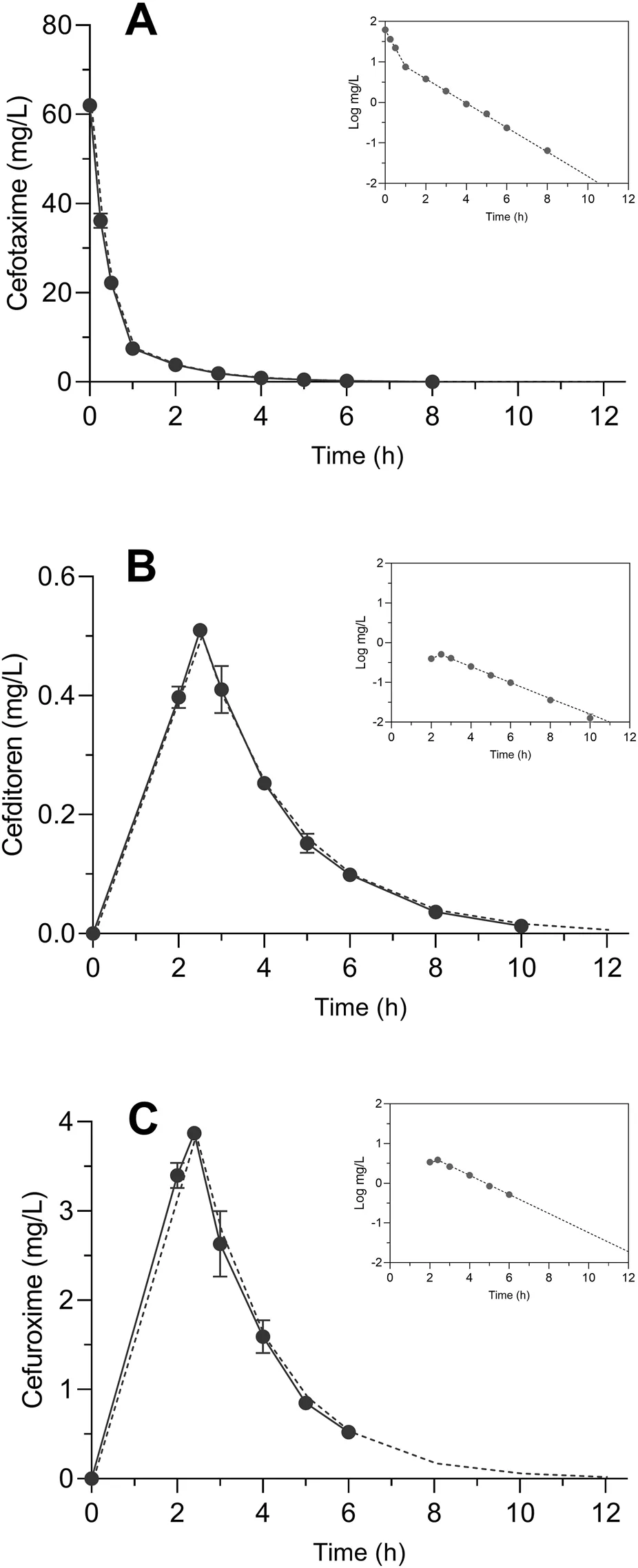
Mean concentration-time profile following single-dose administration in the *in vitro* system. **(A)** cefotaxime, **(B)** cefditoren, **(C)** cefuroxime. The insert in panels depicts log-linear concentration-time profile. The solid circles represent the measured drug levels. The dashed line represents the predicted free drug levels obtained through non-compartmental analysis, based on the target pharmacokinetic (PK) parameters.

Experimental single-dose concentrations for CTX, CDN, and CXM are depicted in Figure 1. Table 2 shows target and observed pharmacokinetic parameters. No significant differences were found between target and experimental pharmacokinetic parameters in uninfected bioreactors (p ≥ 0.12; percent error <3.5%, %CV ≤ 6.0%). Furthermore, drug concentrations exposed to bacteria during infected simulations did not differ from single-dose experiments (p ≥ 0.24), maintaining an overall system prediction error below 4% across all tested regimens (Supplementary Table S1).

**FIGURE 2:**
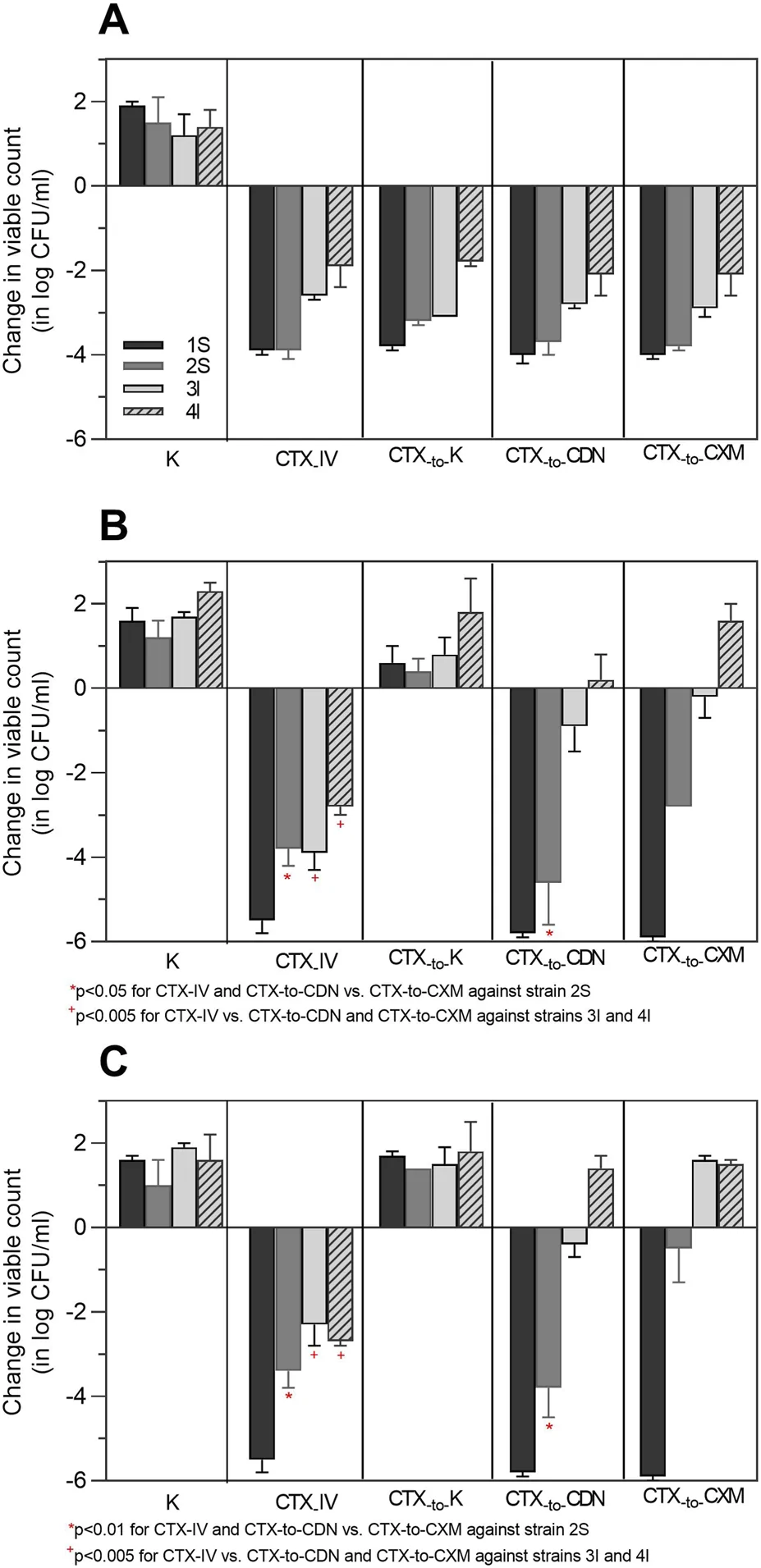
Antibacterial effect (change in viable count, in log CFU/mL) observed at 12 h **(A)**, 24 h **(B)**, and 36 h **(C)** for the experimental dosing regimens against four *S. pneumoniae* strains (1S, 2S, 3I, and 4I). K; growth control (distilled water q12h x3). CTX-IV; cefotaxime IV 1,000 mg q12h x3. CTX-to-K; cefotaxime IV 1,000 mg to distilled water q12h x2. CTX-to-CDN; cefotaxime IV 1,000 mg to cefditoren oral 400 mg q12h x2. CTX-to-CXM; cefotaxime IV 1,000 mg to cefuroxime oral 500 mg q12h x2. Bars represent mean values, and error bars indicate standard deviation (SD).

The mean change in bacterial counts (in log CFU/mL) at 12, 24 and 36 h are shown in Figure 2. The initial count, ranging from 7.28 ± 0.14 to 7.43 ± 0.12 log CFU/mL, increased 1 to 2 log throughout the experiment in the absence of antibiotic (K). The first dose of CTX exerted rapid bactericidal activity against strains 1S and 2 S and showed a mean kill of 2.64 and 1.95 log CFU/mL at 12 h from starting inoculum for strains 3I and 4I, respectively. As shown in Figure 2, at 12 h, after completion of the first IV CTX dose, the initial bacterial density at the time of switching to K, CDN, or CXM (or continued CTX) was not significantly different among the strains (p ≥ 0.26).

The switch from CTX to distilled water (CTX-to-K) caused complete regrowth in all four strains, matching bacterial growth kinetics in the absence of antibiotics (K) at 36 h (Figure 2). In contrast, the switch from CTX to an oral cephalosporin produced different antimicrobial effects depending on the cephalosporin and the strain.

Against strain 1S, both oral cephalosporins sustained bactericidal activity. The mean colony counts fell below the detection limit from 14 h onwards with the CTX-to-CDN and CTX-IV regimens, and from 18 h onwards with CTX-to-CXM.

Against strain 2S, the CTX-to-CDN regimen maintained bactericidal activity throughout. The mean log CFU/mL reduction at 36 h for CTX-to-CDN was equivalent to CTX-IV reference, with no statistically significant differences between them (Figure 2). Conversely, the switch to CXM led to marked bacterial regrowth from 16 h onwards, reaching initial inoculum levels at 36 h (p ≤ 0.001 vs. CTX-IV and CTX-to-CDN regimens.

Against strains 3I and 4I, regrowth occurred with both CTX-to-CDN and markedly with CTX-to-CXM (Figure 2). Against strain 3I, the mean log CFU/mL at 36 h with CTX-to-CXM was not significantly different from the growth control. CTX-to-CDN maintained some activity (0,4-log reduction). Against strain 4I the mean log CFU/mL at 36 h for CTX-to-CDN or CTX-to-CXM was not significantly different from bacterial growth kinetics in the absence of antibiotics (K).

Against strains 3I and 4I, the clinical reference regimen (CTX-IV) was significantly more effective than both sequential regimens at the end of the experiment (p < 0.005 vs. CTX-to-CDN and CTX-to-CXM at 36 h). This regimen sustained the antibacterial effect achieved after the first dose until the end of the study, although it did not reach bactericidal activity (≥3 log reduction) and showed a slight increase in bacterial density for strain 3I at 36 h.

## 4 Discussion

The present simulation was aimed at exploring the *in vitro* PK/PD basis for choosing an oral cephalosporin for sequential therapy following IV CTX treatment against *S*. *pneumoniae*, using the sustained bactericidal/bacteriostatic effect of CTX-IV regimen as the benchmark for therapeutic success. To bridge the gap between *in vitro* assay susceptibility data and real-world clinical application, all simulations were performed exclusively using dosing regimens—doses and dosing intervals—approved for human use.

Overall, against the CTX-susceptible strains according to EUCAST (1S and 2S) the sequential regimen of CTX-to-CDN successfully matched the efficacy of the reference CTX-IV regimen, maintaining the initial bacterial reduction over the 36 h period. In contrast, the CTX-to-CXM regimen failed to sustain the effect of the reference regimen, resulting in pronounced regrowth during oral cephalosporin exposure against strain 2S (categorized as CXM-susceptible by CLSI but resistant by EUCAST). By 36 h, bacterial counts for CTX-to-CXM reached the initial inoculum levels, marking a significant difference compared to both the CTX-IV reference and the CTX-to-CDN regimen (p < 0.01).

Regarding CTX-intermediate strains according to EUCAST (3I and 4I), while the CTX-to-CDN regimen retained some activity (a 0.4 log CFU/mL decrease at 36 h) against strain 3I (MIC_CDN_; 0.25 mg/L), but not against strain 4I (MIC_CDN_; 0.5 mg/L), the CTX-to-CXM regimen consistently showed regrowth. The CTX-IV treatment maintained a sustained bacteriostatic effect against these strains over the experiment. However, while less pronounced, strain 3I exhibited a regrowth pattern like that observed with CTX-to-CDN during the 24–36-h treatment period. In this context, an inoculum effect was ruled out based on the baseline susceptibility values across all tested strains, confirming that the antibacterial activity and regrowth dynamics observed in this model were exclusively driven by the underlying PK/PD relationships of the simulated regimens.

Clearly, the findings for the CTX-to-CXM regimen align more closely with the oral CXM susceptibility interpretation of EUCAST (strains 2S, 3I and 4I resistant) than with CLSI criteria (strain 2S susceptible, 3I intermediate, and 4I resistant). For the CTX-IV regimen, although the activity observed against all strains was at least bacteriostatic—consistent with their classification as susceptible by CLSI—the antibiotic behavior was not homogeneous across the four strains, being therefore more consistent with the susceptible/intermediate distinction established by EUCAST. Regarding cefditoren, since no clinical breakpoints have been established by EUCAST or CLSI for *S. pneumoniae*, its evaluation was strictly based on absolute MIC values and observed pharmacodynamic kill kinetics rather than categorical susceptibility interpretations.

The selected strains (two CTX-susceptible and two CTX-intermediate according to EUCAST) reflected the distribution reported in a recent longitudinal surveillance study in Spain, in which 51.2% of pneumococci were susceptible, 47.9% intermediate, and only 1% resistant to CTX by EUCAST (MIC≥4 mg/L) (Sempere et al., 2022a). Of note, according to the latest CDC data, <1.7% of pneumococci exhibit a MIC_CTX_≥2 (Centers for Disease Control and Prevention, 2023). In that Spanish surveillance, MIC_50_/MIC_90_ of CTX was 0.5/2 mg/L, and it was reported a constant decreasing pattern of reduced susceptibility to penicillin and CTX from 2004 on after introduction of conjugate vaccines (Sempere et al., 2022b). Notably, strain 4I in our study belonged to serotype 11A, a serotype that has shown an increase in recent years, likely due to its absence in PCV13 (Pérez-García et al., 2024; Sempere et al., 2022b); however, a downward trend could be anticipated following its inclusion in PCV20, PCV21 and PPV23 vaccines.

Stringent conditions were intentionally implemented in this study to assess the antimicrobial activity of simulated sequential therapy across multiple clinical scenarios, ranging from the most favorable expected situations (CTX-susceptible strains) to the most complex ones (multidrug-resistant strains with intermediate resistance to CTX). The experimental rigor was further enhanced by exposing bacteria to theoretical free plasma concentrations, extrapolated from total concentrations using the percentage of protein binding. While this approach has a limited impact on the antimicrobial activity of agents with low plasma protein binding (Cafini et al., 2007; Echeverría et al., 2007) as CTX or CXM in this study, its effect is dramatic on agents like CDN, which exhibit high plasma protein binding (Echeverría et al., 2007; Sevillano et al., 2007). In this respect, previous works by our group have shown that the activity of CDN in the presence of total concentrations and albumin/serum in the medium is demonstrably superior to that observed using only free concentrations, thereby suggesting that protein binding is not an absolute limitation (Sevillano et al., 2008; Sevillano et al., 2007). Interestingly, using a similar *in vitro* system but employing human serum as the culture medium, we demonstrated that a theoretical *f*T_>MIC_≈24% was sufficient to achieve bactericidal activity against strains with a MIC_CDN_ = 0.25 mg/L (Sevillano et al., 2008). Furthermore, a theoretical *f*T_>MIC_≈1.7% was related to bacteriostatic effects, with growth reductions ranging from 53% to 97% against strains with a MIC_CDN_ = 0.5 mg/L (Sevillano et al., 2008). The *f*T_>MIC_ for CDN required to confer bactericidal activity would be even lower in preimmunized subjects, as previously demonstrated in a mouse model (Cafini et al., 2010).

It is important to note that although free serum concentrations may not fully reflect local lung compartment dynamics, such as epithelial lining fluid (ELF) penetration, they remain the standard and most validated surrogate marker for predicting antimicrobial efficacy (Drusano et al., 1998). While available data suggest that, unlike cefuroxime, cefditoren achieves favorable penetration into ELF with exposure levels exceeding free serum concentrations (Lodise et al., 2008), current ELF pharmacokinetic datasets lack the methodological robustness required to reliably characterize dynamic time-course profiles for *in vitro* simulation. Therefore, utilizing validated free serum profiles provided the most scientifically sound approach while imposing an intentionally stringent condition for cefditoren.

Even in these expressly unfavorable circumstances, the simulation of sequential therapy using CDN showed no differences compared to the complete CTX-IV regimen against CTX-susceptible strains by EUCAST. Against tested strains with intermediate susceptibility to CTX by EUCAST, the sequential therapy with CDN, as the complete CTX-IV regimen, was bacteriostatic against strain 3I. Based on previous studies exploring the influence of protein binding on CDN activity, *in vivo* in mice and *in vitro* by reproducing the physiological conditions of plasma protein binding (Cafini et al., 2010; Sevillano et al., 2008), it could be hypothesized that under physiological conditions, the exposure to total CDN concentrations would result in greater reductions in bacterial load against CTX-intermediate strains with MIC_CDN_<1 mg/L.

Caution should be exercised when inferring susceptibility to oral cephalosporins from CTX susceptibility (Murphy et al., 2021). The selection of an oral cephalosporin for IV-to-oral sequential therapy must be based on the specific susceptibility of the infecting isolate to the oral cephalosporin to ensure sustained therapeutic efficacy. At this point, potential discrepancies between susceptibility test interpretative criteria (e.g., CLSI vs. EUCAST) should be considered, as they may influence the relative conservativeness of the selected approach. This was evident in the present study, where all four strains were classified as susceptible by CLSI, while EUCAST classified two of them as intermediate; the heterogeneous behavior observed among strains with the CTX-IV simulated regimen was more consistent with the EUCAST classification, reinforcing its more conservative approach. However, since the infecting pathogen could not be isolated in more than 50% of cases (Jain et al., 2015), the results of the present simulation acquire particular importance. For patients without an isolated pathogen who show a favorable response to initial IV cephalosporin therapy (presumed susceptibility to CTX), an inappropriate choice of oral step-down agent could lead to clinical deterioration. This risk is illustrated in our study, where switching to CXM led to bacterial regrowth in strain 2S, despite its documented susceptibility to CTX. This reinforces that the assumption that susceptibility to one β-lactam predicts susceptibility to other agents within the same class is incorrect (Murphy et al., 2021) and suggests that selecting the compound with the highest *in vitro* activity is the most prudent approach to ensure optimal therapeutic outcomes. Among available oral cephalosporins, cefditoren consistently exhibits the lowest MIC values and highest intrinsic *in vitro* activity against *S. pneumoniae*, including penicillin- and cephalosporin-non-susceptible isolates, as demonstrated in longitudinal surveillance studies (Sempere et al., 2022a). Consequently, it has been noted as a suitable agent for switching from IV-to-oral therapy when the initial treatment is an IV third-generation cephalosporin (Cantón et al., 2022; Giuliano et al., 2023; Rodríguez-Leal et al., 2024).

Animal infection models represent the most appropriate strategy to reproduce the physiological complexity of infection, including host immune defenses, protein binding and tissue dynamics. However, utilizing animal models would require highly virulent isolates, whether susceptible or resistant. Such strains are often not representative of typical clinical infections managed with CTX, potentially introducing selection bias. Therefore, given these methodological constraints, we opted to employ a translational *in vitro* system. Although this system offers the best *in vitro* evidence to guide the selection of oral cephalosporins for sequential therapy following IV CTX treatment, it is important to acknowledge certain inherent methodological limitations. First, it remains an *in vitro* simulation. While it represents the most optimal *in vitro* approach to address the problem, it may not fully reflect clinical outcomes for CAP. Second, the study duration was short (36 h), as opposed to the clinical treatment period of 5–7 days for CAP. Third, only one IV dose was administered in our model instead of the standard clinical regimen; this was done to preserve quantifiable microorganism densities at the beginning of the subsequent oral treatment phase. Using the full clinical IV duration would have reduced bacterial counts below the limit of quantification before the oral phase began, precluding meaningful comparison between regimens. While this design does not reproduce the exact duration of clinical IV therapy, it reflects the same underlying principle of clinical step-down strategies, in which the switch to oral treatment occurs once the bacterial burden has already been substantially reduced. If anything, preserving a higher starting inoculum than would typically remain after a full course of IV therapy represents a more stringent test of the oral regimens rather than a bias in their favor. Finally, the isolates used in the study may not represent the full spectrum of behavior exhibited by all pneumococcal strains. Additionally, incorporating population PK/PD modeling through Monte Carlo simulations would complement our experimental findings. This approach would help evaluate potential target attainment and optimize oral sequential regimens against regional MIC distributions.

In conclusion, in this *in vitro* pharmacodynamic study conducted under stringent simulated conditions, CDN showed greater antibacterial activity than CXM during the simulated transition from IV to oral therapy and achieved bacterial reductions comparable to continued IV treatment against the CTX-susceptible strains. Furthermore, the results should be interpreted considering the intrinsic limitations of *in vitro* models, including the free-drug assumptions used to simulate CDN exposure, the shortened duration of treatment simulated, the MIC range and the limited number of strains tested. Confirmation in clinical studies is warranted before any therapeutic recommendation can be made.

## Data Availability

The raw data supporting the conclusions of this article will be made available by the authors, without undue reservation.
