# Supplementary material for: Simulating an intravenous-to-oral cefotaxime-to-cefditoren/cefuroxime switch: an in vitro pharmacodynamic approach against selected Streptococcus pneumoniae strains with defined susceptibility profiles
Source: Front Pharmacol. 2026 Aug 24;17:1770715. doi: 10.3389/fphar.2026.1770715 (PMC13547826; doi:10.3389/fphar.2026.1770715)
Supplement: Supplementary file 1 [file Supplementaryfile1.docx]

**Supplementary material**

**Figure S1.** In vitro two-compartment PK/PD system

Components are as follows: 1 central flask; 2, dilution reservoir (nutrient broth); 3, collection vessel; 4, peristaltic pumps; 5, injector (infusion pump); 6, polytetrafluoroethylene (PTFE) tubing; 7, silicone tubing; 8, bioreactor; 9, inoculation port; 10, sample port; 11, RS-232 connection; 12, Gilson serial input/output channel (GSIOC) connection; 13, temperature probe; 14, air filter.

The nutrient broth was pumped from the dilution reservoir to the central flask at a flow rate designed to simulate the half-life of the cephalosporins under study. The content of the central flask was then circulated through the hollow fiber of the bioreactor at a rate of 50 ml/min, with an additional recirculation flow of 25 ml/min within the extracapillary space of the bioreactor. To maintain a constant volume of distribution of 350 ml throughout the system, the central flask was connected to a collecting vessel for overflow.

**Table S1:** Pharmacokinetic validation of observed vs. target concentration in infected bioreactors

| **Antibacterial agent**^a^ | **Sample time**  **(h)** | ***n*** | **Observed *C* (mg/L)**^b^ | **Target *C***  **(mg/L)^c^** | **%Error**^d^ | **Target *C* in uninfected bioreactors (mg/L)**^e^ | **%Error**^d^ |
| --- | --- | --- | --- | --- | --- | --- | --- |
| CTX | 0.25 | 32 | 37.24 ± 0.74 | 36.37 | 2.71 | 36.15 | 3.19 |
|  | 12.25 | 8 | 36.8 ± 1.24 | 36.37 | 2.89 | 36.15 | 2.94 |
|  | 18 | 8 | 2.89 ± 0.01 | 0.239 | 3.31 | 0.235 | 3.37 |
|  | 24.25 | 8 | 36.62 ± 1.27 | 36.37 | 3.03 | 36.15 | 3.05 |
| CDN | 14.5 | 8 | 0.511 ± 0.013 | 0.5 | 2.65 | 0.51 | 1.96 |
|  | 18 | 8 | 0.097 ± 0.004 | 0.099 | 3.65 | 0.1 | 3.61 |
|  | 26.5 | 8 | 0.505 ± 0.012 | 0.5 | 1.98 | 0.51 | 1.79 |
| CXM | 14.4 | 8 | 3.82 ± 0.14 | 3.85 | 2.95 | 3.87 | 2.88 |
|  | 18 | 8 | 0.504 ± 0.019 | 0.502 | 2.94 | 0.52 | 3.46 |
|  | 26.4 | 8 | 3.9 ± 0.12 | 3.85 | 2.99 | 3.87 | 3.23 |

^a^ CTX (cefotaxime); spot samples collected across all CTX-containing regimens (CTX-IV, CTX-to-K, CTX-to-CDN and CTX-to-CXM). CDN (cefditoren); spot samples collected during the oral phase of the CTX-to-CDN regimen. CXM (cefuroxime); spot samples collected during the oral phase of the CTX-to-CXM regimen.

^b^ Observed Concentration: Experimental drug concentration measured directly from the infected bioreactor chamber during the 36-hour dynamic simulations, expressed as mean $\pm$ standard deviation (SD) of independent runs

^c^ Target concentration: Nominal free drug concentrations predicted by mathematical modelling based on simulated human pharmacokinetic parameters.

d Percent error, calculated as │*C*_infect_-*C*_target_│/*C*_target_ *100

e Target concentration in uninfected bioreactors: Mean experimental free drug concentrations measured during preliminary single-dose baseline runs in uninfected bioreactors.

n; Number of independent biological spot-samples collected from infected bioreactors and Number of quantified in triplicate by bioassay (total N=104).
